# Homogeneous Selection and Dispersal Limitation Dominate the Effect of Soil Strata Under Warming Condition

**DOI:** 10.3389/fmicb.2022.801083

**Published:** 2022-02-24

**Authors:** Zhujun Wang, Kai Feng, Guangxin Lu, Hao Yu, Shang Wang, Ziyan Wei, Ning Dang, Yingcheng Wang, Ye Deng

**Affiliations:** ^1^CAS Key Laboratory of Environmental Biotechnology, Research Center for Eco-Environmental Sciences, Chinese Academy of Sciences, Beijing, China; ^2^College of Resources and Environment, University of Chinese Academy of Sciences, Beijing, China; ^3^College of Agriculture and Animal Husbandry, Qinghai University, Xining, China; ^4^College of Environmental Science and Engineering, Liaoning Technical University, Fuxin, China; ^5^State Key Laboratory of Microbial Resources, Institute of Microbiology, Chinese Academy of Sciences, Beijing, China

**Keywords:** global warming, soil strata, assembly process, Tibetan grassland, homogeneous selection, dispersal limitation

## Abstract

Global warming is likely to affect the underground microbial communities in various ecosystems, but the response of soil microbial communities along a vertical depth profile to global warming has been elusive. Herein, we leveraged a warming field experiment in Qinghai-Tibet Plateau grassland and investigated the community structure of prokaryotes and fungi from the upper (0–15 cm) and lower (15–30 cm) strata under ambient and elevated temperature treatments. Three-years continual warming only significantly shifted the prokaryotic community within the upper strata and there was no significant effect observed for the fungal community. Additionally, under ambient temperature, there were significant differences between the two strata in both the prokaryotic and fungal communities, but under warming, this effect was alleviated. Next, the prokaryotic and fungal community assembly processes were measured by a phylogenetic-bin-based null approach (iCAMP). Though deterministic and stochastic processes dominated the assembly of prokaryotic and fungal communities, respectively, the deterministic processes were strengthened under warming for both communities. Specifically, the increased portion of homogeneous selection, contributing to a homogenous state, led to a smaller difference between prokaryotic communities of the two soil strata under warming. The smaller difference in the stochastic process component, *i.e.*, dispersal limitation, contributed to the similar fungal community structures between the two strata under warming. This study deepens our understanding of warming effects on grassland microbial communities and gives greater insights on the underlying mechanisms for microbial assembly between upper and lower soil strata under warming scenarios.

## Introduction

The average temperature over land for the period 2006–2015 was 1.53°C higher than for the period 1850–1900, and 0.66°C larger than the equivalent global mean temperature change reported by IPCC (2019) and Shukla et al. (2019). The Qinghai-Tibet Plateau is one of the most sensitive and threatened regions under climate warming (Liu and Chen, 2000; Kang et al., 2010). This region is predicted to experience greater than average increases in surface temperatures and will likely be reduced to 20% of its current area by the end of the 21st century due to climate change (Walker et al., 2001; Yang et al., 2014). Moreover, the Tibetan Plateau harbors 2.5% of the global soil C pool and may undergo significant release of C due to climate warming (Wang et al., 2002). There is extensive evidence showing that climate warming has a strong effect on soil microbial community composition and function in the Tibetan alpine ecosystem (Zhao et al., 2014; Rui et al., 2015; Liu et al., 2016; Zhang et al., 2016). Therefore, it is important to understand the potential feedback of the microbial community under future climate change scenarios; however, we still lack the underlying mechanisms of these responses in Tibetan alpine grassland.

Variations of soil structure across strata are correlated with changes in microbial communities (Fierer et al., 2003; Du et al., 2021). The changes in microbial community composition across strata are important for understanding microbial community assembly processes (Zhou and Ning, 2017; Liu et al., 2021). The relative importance of the deterministic and stochastic processes on soil microbial communities varies across environmental gradients (e.g., pH, nutrients, and climate), succession stages, and different functional assemblages (Liu et al., 2021). Soil microbial communities are controlled by deterministic processes more than by stochastic processes in topsoils (0–10 cm) and the deterministic factors are mainly climate-related factors (Hanson et al., 2012) or also oligotrophic environments in subsoils related to soil geochemical factors (i.e., soil pH) (Tripathi et al., 2018). Previous studies have shown that the subsoil may respond differently to global climate changes than topsoil due to the distinct soil environments, microbial assemblages, and their functional responses to climate changes (Cheng et al., 2017; Jia et al., 2017; Li et al., 2018; Liu et al., 2019). However, less is known for soil strata, especially with warming effects on microbial communities, in alpine grasslands.

Prokaryotic and fungal communities respond differently to warming in soil. Prokaryotes, in general, seem to be more sensitive to warming, responding in a positive manner in top soil (Sheik et al., 2011; Zhou et al., 2016; Dennis et al., 2019; Guo et al., 2019; Kanzaki and Takemoto, 2021), while fungi are more tolerant (Zhang et al., 2016, 2020; de Oliveira et al., 2020). These incongruent responses of prokaryotes and fungi within different strata to warming and their assembly mechanisms in alpine grassland are still not well understood.

In this study, we aimed to explore the microbial communities of prokaryotes and fungi from upper and lower soils under ambient and elevated temperature treatments in a Qinghai-Tibet Plateau grassland. We hypothesized that (i) microbial communities would be different between soil strata in Tibetan alpine grasslands, and these responses would be altered by warming effect; (ii) these different microbial responses may due to different microbial assembly mechanisms. To test our hypotheses, we utilized a 3-year warming experiment on Tibetan alpine grassland’s microbial communities, from 0 to 30 cm, and their responses to the interactive effects of warming between different soil strata. Our observations provided evidence toward a mechanistic understanding of how the soil microbial community could change between different soil strata under future global warming conditions.

## Materials and Methods

### Site Description, Experimental Design, and Soil Sampling

Warming and N amendment experiments were carried out at the Sanjiangyuan Alpine Grassland Ecosystem Field Observation Station (N33°24′30″, E97°18′00″, altitude 4,270 m) on Southern Tibetan Plateau, Qinghai, China. This area is an alpine grassland, dominated by *Kobresia pygmaea, Lagotis ramalana, Potentilla nivea*, and *Thalictrum alpinum* (Wen and Lu, 2015; Wang et al., 2019). As described previously (Wang et al., 2019), this site is characterized by a continental monsoon climate, comprised of cold (October to May) and growing (June to September) seasons, with an annual mean air temperature from -5.6 to 3.8°C during 1985–2004, and annual average precipitation of 562.2 mm, with ∼75% of annual precipitation occurring during the growing season.

Experimental plots were set up in July 2013, in which terrain was flat and plant community composition was relatively uniform. It was surrounded by an enclosure to avoid the grazing disturbance. Warming experiments were carried out using 4 m^2^ × 0.4 m tall Open-top chambers (OTCs) (Henry and Molau, 1997), constructed with clear polyvinyl chloride (PVC) and a steel framework (Wang et al., 2019). OTCs increased mean near-surface temperatures by 2.00 ± 0.24°C. Meanwhile, either ammonium sulfate or sodium nitrate was amended as the external N source for alpine grassland at 300 kg N ha year^–1^ for maximal plant biomass (Guo et al., 2014). Soil samples were collected from the 0–15 to 15–30 cm strata, due to the majority of grass root growing between 0 and 15 cm. As our focus was on the effects of warming on different soil strata, we set group treatments, including ammonium sulfate and sodium nitrate amendment, with nine replicates per group treatment as: (i) warming upper (WUp), warming treatment at 0–15 cm strata; (ii) warming lower (WLo), warming treatment at 15–30 cm strata; (iii) unwarming upper (UWUp), ambient temperature at 0–15 cm strata; and (iv) unwarming lower (UWLo), ambient temperature at 15–30 cm strata.

Soil samples were collected in August 2016. Soil cores, 5 cm in diameter, were taken from 0 to 30 cm at three randomly chosen locations within each 4 m^2^ plot and divided into 0–15 and 15–30 cm layers, then mixed together, respectively, to form composite samples. Soil samples were set on ice then transported back to the laboratory where visible grassroots and pebbles were subsequently removed. Soil samples were stored at −80 and 4°C for DNA extraction and soil geochemical measurements, respectively. Soil geochemical measurements, including pH, ammonium and nitrate content, total nitrogen content, organic matter, available nitrogen content, soil temperature, and soil moisture were described previously (Wang et al., 2019).

### DNA Extraction, Polymerase Chain Reaction Amplification, Sequencing, and Bioinformatics Processing

Genomic DNA was extracted from 2 g of soil with the freeze-grinding method as described previously (Zhou et al., 1996), and purified using the PowerSoil ^®^ DNA Isolation Kit (MoBio Laboratories, Inc., Solana Beach, CA, United States) according to the manufacturer’s instructions. For polymerase chain reaction (PCR) amplification, DNA quality and concentration were assessed based on absorbance ratios of 260/280 nm (∼1.8) and 260/230 nm (>1.7) as detected by a NanoDrop Spectrophotometer (Nano-100, Aosheng Instrument Co. Ltd., Hangzhou, China). For Illumina sequencing, we used PicoGreen ^®^ DNA Quantitation Assay (Thermo Fisher Scientific Inc., Waltham, MA, United States) and Qubit Fluorometric Quantitation (Thermo Fisher Scientific Inc., Waltham, MA, United States) to quantitate DNA amounts.

For bacterial and archaeal 16S rRNA genes, the V4 region was amplified with the primer pair 515F (5′-GTGCCAGCMGCCGCGGTAA-3′) and 806R (5′-GGACT ACHVGGGTWTCTAAT-3′) (Caporaso et al., 2011); for ITS genes, the ITS2 region was amplified with the primer pair gITS7F (5′-GTGARTCATCGARTCTTTG-3′) and ITS4R (5′-TCCTCCGCTTATTGATATGC-3′) (Ihrmark et al., 2012), combined with self-designed barcodes to distinguish samples. The details of PCR amplification, amplicon purification, library preparation, Illumina MiSeq sequencing, and sequence processing were described previously (Feng et al., 2017; Wei et al., 2018). Sequence processing was conducted on an in-house pipeline^1^ integrated with the necessary bioinformatics tools (Feng et al., 2017; Wei et al., 2018). UPARSE (Edgar, 2013) was used to remove chimeras and classify the sequences into operational taxonomy units (OTUs) at a similarity of 97% with singletons being discarded. A randomly resampled OTU table was obtained to normalize total reads with a resample size of 24,084 reads for 16S rRNA gene and 10,253 reads for ITS gene based on the minimum sequence number per sample. The sequencing data are available in National Microbiology Data Center^2^ with project number NMDC10017898.

### Biodiversity and Statistics Analysis

The biodiversity indices for α-diversity and β-diversity were evaluated for both prokaryotic and fungal groups. The Shannon index, inverse Simpson index, observed richness, and Faith’s phylogenetic diversity were estimated to indicate the microbial species diversity. Based on weighted UniFrac distance matrix, principal coordinate analysis (PCoA) was used to show the difference among the prokaryotic or fungal communities from upper and lower soils under ambient and elevated temperature treatments, and the significance for warming and strata contributions to the microbial community structure were analyzed by permutational multivariate analysis of variance (PERMANOVA). Moreover, linear discriminant analysis effect size (LEfSe) was used to find the specialists for the different communities at the OTU level with the criterion of LDA score of 3.0 and significance level of 0.05. Further, the relationship between biodiversity and environmental variables were evaluated with Mantel test and constrained canonical analysis (CCA), and the explained proportion of variance by environmental groups was demonstrated with variance partition analysis (VPA). In addition, one-way ANOVA was used to test the differences of multiple groups. All biodiversity and statistical analyses were conducted with an online in-house pipeline (see text footnote 1; Feng et al., 2017).

In order to quantify the underlying mechanisms of microbial community assembly, we used a newly developed method, called Infer Community Assembly Mechanisms by Phylogenetic-bin-based null model (iCAMP) (Ning et al., 2020). iCAMP assigns the microbial OTUs into phylogenetic-closed groups (bins) and then quantifies the contribution of each ecological process to microbial community assembly, including homogeneous selection (HoS), heterogeneous selection (HeS), dispersal limitation (DL), homogenizing dispersal (HD), and undominated (DR). Afterward, the proportion of the ecological process between different samples can be compared based on the bootstrapped methods, and the contribution of each phylogenetic bin can be identified as well. In addition, another approach, normalized stochasticity ratio (NST), was used to determine the relative importance of determinism and stochasticity for microbial community assembly (Ning et al., 2019). The analyses were conducted with the “iCAMP” (v. 1.3.4) and “NST” (v. 3.0.6) packages in R (v. 4.1.1).

## Results

### Soil Physiochemical Variables and Microbial Communities Showed Differences Across Temperature and Vertical Soil Profiles

As our previous study showed (Wang et al., 2019), after 3 years of warming treatment, the soil moisture had increased. Warming also significantly increased mean plant height and plant biomass. Warming increased soil mean annual temperature 1.40 and 1.16°C at upper and lower soil strata, respectively; while it made no difference between upper and lower soil strata (Supplementary Figure 1). However, it did not alter other soil properties (Supplementary Figure 1) or alpha diversities of the prokaryotic (Supplementary Figure 2) and fungal communities (Supplementary Figure 3). Meanwhile, there were significant differences in soil properties between the upper and lower soil strata (Supplementary Figure 1), as well as alpha diversity of the prokaryotic (Supplementary Figure 2) and fungal communities (Supplementary Figure 3). Soil ammonium and nitrate content, total and available nitrogen content, and organic matter were significantly higher in the upper strata, while pH was the only property that was significantly lower in the upper strata. Alpha diversities of the prokaryotic community were higher in the upper soil, while those of the fungal community were higher in the lower soil strata.

The prokaryotic and fungal community compositions were significantly different between soil strata under unwarming treatment as revealed by PERMANOVA and PCoA (Table 1 and Supplementary Figure 4). However, microbial community compositions of the two soil strata were similar under warming treatment.

**TABLE 1:** Effects of warming and strata on microbial communities.

Profiles	Comparison group	Prokaryote		Fungi	
			
		*F*	*p*	*F*	*p*
Warming	UWUp vs. WUp	1.9423	0.048*	0.6372	0.499
	UWLo vs. WLo	1.6999	0.108	1.6792	0.109
Strata	UWUp vs. UWLo	3.1822	0.001***	3.1965	0.007**
	WUp vs. WLo	1.2253	0.221	0.5250	0.672

***p < 0.001, **p < 0.01, *p < 0.05, based on permutational multivariate analysis of variance (PERMANOVA). UWUp, unwarming upper; UWLo, unwarming lower; WUp, warming upper; WLo, warming lower. The bold values indicated P < 0.05.

### Fewer Prokaryotic and Fungal Specialists Were Observed Under Warming Conditions

**FIGURE 1:**
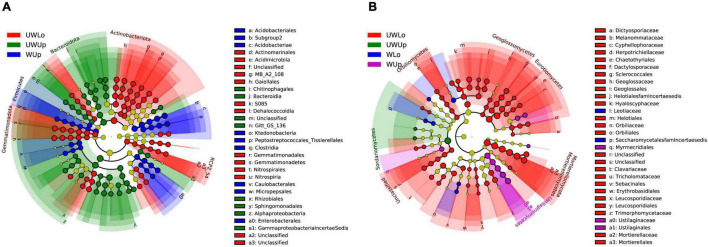
Linear discriminant analysis effect size (LEfSe) analysis identified the most differentially abundant taxa among treatments (*p* < 0.05; LDA score 3.0) based on 16S rRNA gene amplicon sequences **(A)** and ITS gene amplicon sequences **(B)**. Differences are represented in the color of the most abundant class, while no significant difference is indicated by yellow. The diameter of each circle is proportional to the abundance of the taxon, representing the kingdom, phylum, class, order, family, genus, and species. UWUp, unwarming upper; UWLo, unwarming lower; WUp, warming upper; WLo, warming lower.

Based on the phylogenetically informed classification results from RDP classifier, linear discriminant analysis (LDA) effect size (LEfSe) was utilized to identify potential specialist taxa that were different between the two strata with warming. LEfSe identified 16 prokaryotic taxa above order level from unwarming lower soil, nine from unwarming upper soil, 10 from warming upper soil (Figure 1A and Supplementary Figure 5A). In addition, LEfSe identified 31 fungal taxa above family level from unwarming lower soil, one from unwarming upper soil, two from warming lower soil, and four from warming upper soil (Figure 1B and Supplementary Figure 5B). For the prokaryotic community, Gemmatimonadota and Actinobacteriota were more enriched in the unwarming lower soil, Alphaproteobacteria and Bacteroidota in the unwarming upper soil, while Firmicutes was more enriched in the warming upper soil (Figure 1A and Supplementary Figures 5A, 6A). Within the fungal community, Mortierellomycota and Eurotiomycetes were more enriched in the unwarming lower soil, Sordariomycetes in the unwarming upper soil, while Candida was more enriched in the warming lower soil and Ustilaginomycetes in the warming upper soil (Figure 1B and Supplementary Figures 5B, 6B). The above results indicated that more specialists were only observed under ambient control conditions than the warming treatment, suggesting that warming would reduce the differences between soil strata for prokaryotic and fungal specialists.

### Prokaryotic Community Structure Showed Higher Sensitivity to Environmental Variables

Mantel test results showed that the prokaryotic community was significantly related to all soil properties except ammonium and nitrate content, while the fungal community was only significantly related to total nitrogen content, but with a low *r* value (Table 2). The CCA model was significant for the prokaryotic community when the significant soil measurements were included as independent variables in explaining the variation of the prokaryotic community composition (*p* = 0.001; Supplementary Figures 7A,C), but was not significant for fungal community composition (*p* = 0.067; Supplementary Figures 7B,C). VPA results showed that soil properties and their interactions explained 28.16% of the variation in prokaryotic community composition (Supplementary Figure 7A), and 25.12% of the variation in fungal community composition (Supplementary Figure 7B). These results suggested that the prokaryotic community composition significantly correlated with most soil properties in this Tibetan alpine grassland, while fungal community composition did not. Therefore, the community structure of prokaryotes was more sensitive to environmental variables than fungi. However, soil properties explained only a small portion of the microbial community variation.

**TABLE 2:** Mantel test of environmental factors with microbial communities based on Bray-Curtis distance matrix.

	Prokaryote		Fungi	
		
	*r*	*p*	*R*	*p*
NH_4_-N	0.0933	0.11	−0.1127	0.992
NO_3_-N	0.0812	0.179	−0.0793	0.897
TN	0.2017	**0.004**	0.0991	**0.05**
AN	0.1661	**0.014**	0.0141	0.362
OM	0.2143	**0.009**	0.0895	0.087
pH	0.1827	**0.001**	0.0549	0.14
MAT	0.1634	**0.006**	0.0742	0.059

*NH_4_–N, ammonium content (mg/kg); NO_3_–N, nitrate content (mg/kg); TN, total nitrogen content (g/kg); AN, available nitrogen content (mg/kg); OM, organic matters (g/kg); MAT, soil mean annual temperature.*

*p values shown in bold are at 0.05 significance level.*

### Prokaryotic and Fungal Communities Were Assembled by Different Ecological Processes

In order to explore the relative importance of determinism and stochasticity for microbial community assembly, the null-model-based NST method estimated the normalized stochasticity ratio and found that stochastic processes dominated the microbial assembly for prokaryotes and fungi across the treatments (Supplementary Figure 8). Essentially, the taxonomic stochastic ratio decreased for the prokaryotic community under warming from 79.7–82.9 to 62.5–65.0%, and there was a slightly higher stochastic ratio for the lower soil strata communities compared to upper soil samples (Supplementary Figure 8A). Fungal communities showed higher stochastic ratio with phylogenetic NST as compared with taxonomic NST, and warming also increased the deterministic ratio (<50%, Supplementary Figure 8B). These results suggested both prokaryotic and fungal communities showed less stochasticity under warming treatment and in the upper soils of the alpine grassland.

**FIGURE 2:**
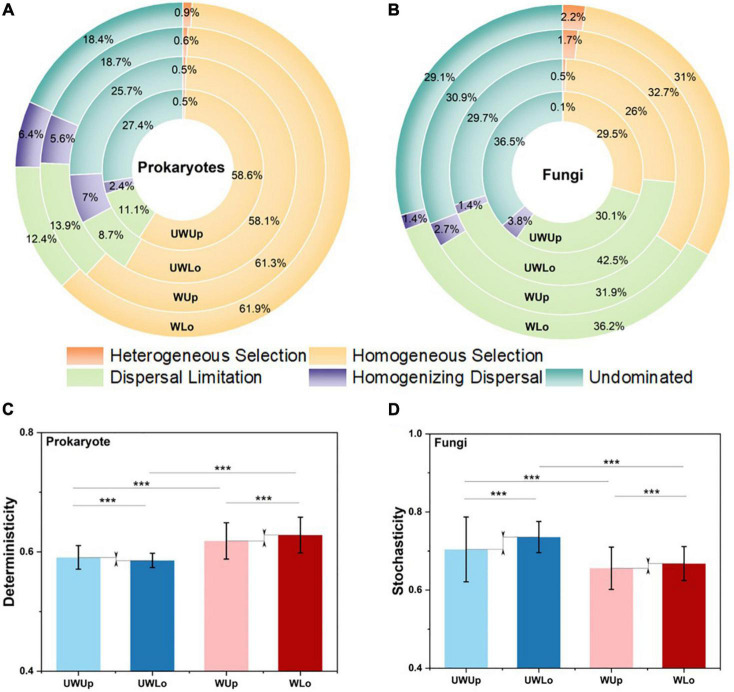
Proportion of ecological processes for prokaryotic **(A)** and fungal **(B)** community assembly. Homogeneous selection and dispersal limitation were selected to make comparison between different communities for prokaryotic **(C)** and fungi **(D)**, respectively. ***p < 0.001, this significance was measured by unpaired Student’s *t* test. UWUp, unwarming upper; UWLo, unwarming lower; WUp, warming upper; WLo, warming lower.

Furthermore, the iCAMP method assigned the deterministic and stochastic processes into five specific ecological processes (Figure 2). Homogeneous selection (58.1–61.9%) and dispersal limitation (30.1–42.5%) dominated the main processes for prokaryotic and fungal communities, respectively, for both warming treatment and strata profiles (Figures 2A,B), indicating higher deterministic components for prokaryotic rather than fungal community assembly. Moreover, warming significantly increased the proportion of the deterministic component (heterogeneous selection and homogenous selection) both for prokaryotic (58.6–59.1 vs. 61.8–62.8%) and fungal (26.4–29.6 vs. 33.2–34.4%) communities within the same strata (Student’s *t* test; *p* < 0.001), showing deterministic tendency under climate warming scenario (Figures 2C,D), especially for the homogenous selection process within the prokaryotic (58.1–58.6 vs. 61.3–61.9%) and fungal communities (26–29.5 vs. 31–32.7%). As for the stochastic component, the proportion decreased in fungi (70.4–73.6 vs. 65.6–66.8% vs.) under warming treatment. Considering strata profiles, the ecological processes showed a distinct tendency within the prokaryotic and fungal communities. For example, the proportion of dispersal limitation increased (31.1–31.9 vs. 36.2–42.5%) and the proportion of homogenous selection decreased (29.5–32.7 vs. 26–31%) within lower soils as compared to upper soils for the fungal community, while the prokaryotic community showed the opposite tendency. More importantly, the difference in stochasticity between upper and lower fungal communities decreased under the elevated temperature condition (65.6 vs. 66.8%) as compared to ambient temperature condition (70.4 vs. 73.6%), and dispersal limitation displayed a similar trend (Figure 2). Thus, the prokaryotic and fungal communities showed different assembly mechanisms between soil strata for ambient and elevated temperature conditions.

### Ecological Processes of Prokaryote and Fungi at Finer Scale

**FIGURE 3:**
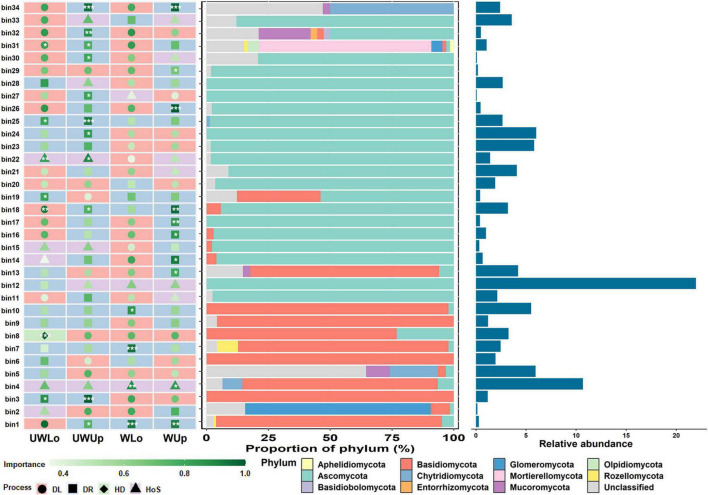
The importance of each phylogenetic bin of the fungal community with associated taxa composition and relative abundance. ***p < 0.001, **p < 0.01, *p < 0.05, showed the significance of the dominating process in each bin based on 1000 bootstrapping times. UWUp, unwarming upper; UWLo, unwarming lower; WUp, warming upper; WLo, warming lower; DL, dispersal limitation; DR, undominated; HD, homogeneous dispersal; HoS, homogeneous selection.

To further understand the underlying mechanisms of such distinct ecological process patterns, we looked into the ecological processes of the dominant groups that may make the greatest contributions to community variance, and the relationships between ecological processes and environmental parameters. First, the representative ecological processes were identified for each bin, which were grouped in closed phylogenetic relations, and the change of ecological process for each bin under different conditions may explain the differences in community assembly for prokaryotes and fungi at a finer scale (Figure 3 and Supplementary Figure 9). The three bins with higher relative abundance within the whole community for prokaryotes were bin27 (11.5%), bin115 (5.8%), and bin72 (4.8%), of which all dominant processes were assigned to homogeneous selection between the two strata and temperature conditions (Supplementary Figure 9). However, the representative processes of the top three fungal bins, i.e., bin12 (22.0%), bin4 (10.7%), and bin24 (6.0%), with high relative abundance changed between the different strata and temperature conditions (Figure 3). The ecological process for bin12 was undominated for unwarming lower soils, while all others were homogeneous selections. Similarly, warming changed the assembly mechanism of bin24, from undominated to dispersal limitation. These variations in status for different bins may be associated with assembly of the whole microbial community. Considering the influence of soil variables on the microbial community, Mantel test was used to correlate the soil heterogeneity with microbial ecological processes (Supplementary Tables 2, 3). Soil nutrient elements (e.g., total nitrogen, available nitrogen, and organic matter) were significantly correlated to homogeneous selection of prokaryotes under ambient temperature condition, and dispersal limitation under elevated temperature condition tended to be associated with nutrient elements as well (Supplementary Table 2). Different from the prokaryotic community, the ecological processes of fungi showed less correlation to environmental variables, but the dispersal limitation was slightly correlated to ammonia nitrogen and organic matter for lower soils at ambient (*r*_mantel_ = -0.341, *p* = 0.0419) and elevated (*r*_mantel_ = 0.421, *p* = 0.0105) temperature conditions, respectively (Supplementary Table 3). The differences in soil nutrient elements between upper and lower soils may be associated with microbial assembly processes, especially the difference for ambient and elevated temperature condition.

## Discussion

**FIGURE 4:**
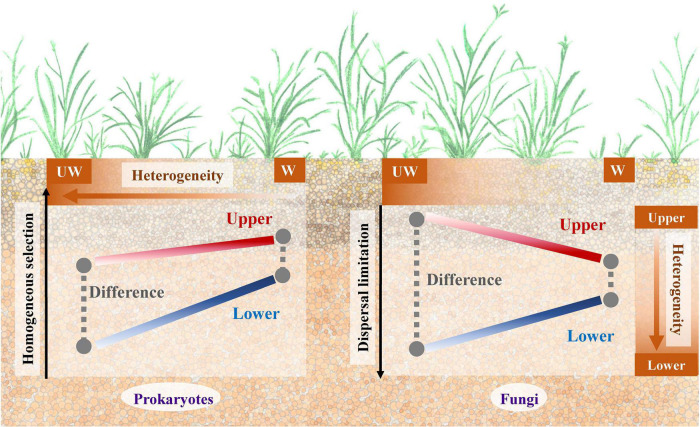
A schematic plot to show the potential underlying mechanisms for the prokaryotic and fungal communities in alpine grassland between strata and temperature profiles. UW, unwarming; W, warming.

As the Qinghai-Tibet Plateau is a region highly sensitive to, and threatened by, climate change (Liu and Chen, 2000; Kang et al., 2010), understanding how microbial communities present in this area respond is extremely important (Yang et al., 2014). In this study, we aimed to elucidate how the microbial community changes between different soil strata under a future warming scenario. For that, we set up an experimental site on Qinghai-Tibet Plateau alpine grassland with both elevated and ambient temperatures and assessed the soil strata between 0–15 and 15–30 cm. After 3-year warming, soil from each treatment was sampled for soil property characterization and 16S rRNA and ITS gene sequencing. A schematic plot (Figure 4) shows the potential underlying mechanisms for prokaryotic and fungal communities in alpine grassland between soil strata under warming scenario.

Similar with our previous study (Wang et al., 2019), warming treatment made no difference, while soil strata made a significant difference for soil properties, except soil mean annual temperature (Supplementary Figure 1) and microbial alpha diversity based on 16S rRNA and ITS gene sequencing data (Supplementary Figures 2, 3). However, fungal community structure was less sensitive to warming treatment compared to the prokaryotic community (Table 1 and Supplementary Table 1). This significant difference between soil strata has been widely studied previously (Liu et al., 2019, 2021; Du et al., 2021), while other possible explanations for the inconsistent results of warming with other studies in Tibetan alpine meadow ecosystems (Xiong et al., 2014; Rui et al., 2015; Li et al., 2016) include the high heterogeneity of soil environments, low taxonomic resolution of the experimental approaches, and/or high noise associated with random sampling (Xue et al., 2016; Wang et al., 2019). However, the prokaryotic and fungal community compositions and structures were significantly different between soil strata under unwarming treatment but more similar under warming treatment (Figure 1, Supplementary Figures 4–6, and Table 1). In addition, the Mantel test, CCA, and VPA results showed that the prokaryote community structure was more sensitive to environmental variables than that of fungi, but the soil properties still explained only a small amount of the microbial community variations (Supplementary Figure 7 and Table 2). The underlying mechanisms of the microbial community responses to warming within different strata require further investigation.

Although fungal community was less sensitive to warming treatment than the prokaryotic community, the difference between upper and lower soils was alleviated by warming for both communities (Table 1). However, the underlying mechanisms for these two communities might be different by considering the microbial assembly processes (Figures 2, 4). In general, homogeneous selection dominated prokaryotic community assembly, while dispersal limitation was a large component for the fungal community. Moreover, homogenous selection of the prokaryotic and fungal communities was promoted in response to warming treatment, consistent with a separate grassland warming field experiment (Ning et al., 2020). Homogenous selection is a type of ecological factor that alters the community structure with a homogenous state, thus resulting in similar community structure with the deterministic variables, e.g., biotic and abiotic conditions (Zhou and Ning, 2017). The correlations showed that the higher heterogeneity was associated with the low proportion of homogenous selection for ambient temperature condition, and fewer correlations were observed for the elevated temperature condition, suggesting similar environmental structures might filter homogenous prokaryotic community, thus decreasing the difference in community structure between upper and lower soils. Different from the prokaryotic community, the dispersal limitation of fungi in upper soils increased as well, but the proportion decreased in lower soils, possibly due to the increase of the homogeneous selection component under warming conditions. Dispersal limitation is considered to be when the migration of an organism is limited due to certain restrictions, e.g., spatial distance and environmental filtering (Zhou and Ning, 2017). The smaller variance of dispersal limitation for fungal community between upper and lower soils under warming condition, may promote the dispersal of fungal species, thus leading to a similar community structure. Interestingly, the dispersal limitation was partially associated with soil nutrient elements in the lower soils, suggesting the dispersal of fungi might be limited by nutrient acquisition, since soil nutrients tend to decline with the strata profile (Tian et al., 2017). Therefore, homogenous selection and dispersal limitation played important roles for the prokaryotic and fungal communities, respectively, with both resulting in similar microbial community structure under warming condition between upper and lower soils (Figure 4).

Climate warming not only changed the microbial community structure in upper grassland soils but also alleviated the differences in the microbial communities along the vertical soil profile, indicating a decreasing β-diversity between the strata. The decreasing β-diversity resulted in a similar community structure and microbial species occupying similar ecological niches within the local ecosystem, which might be vulnerable to disturbance at local scale, since higher β-diversity may provide stability within regional ecosystems (Wang et al., 2016). Thus, we proposed that strata effects (vertical profiles) should be taken into account for microbial community structure variance in response to climate change, especially climate warming. The microbial community structure within lower soils was quite different from the upper soils, and deterministic processes played important roles in these lower regions for the prokaryotic community (Du et al., 2021). Moreover, considering the robustness of fungi to environmental disturbance, as compared to prokaryotes (de Vries et al., 2018), greater variance of the fungal community under climate change might be clearly observed field if experiments are carried out over a long-time span. Additionally, the inter-domain associations or interactions among prokaryotes, fungi, and protists might be reshaped in response to environmental disturbance (Tan et al., 2021; Zhou et al., 2021). In this study, we ignored the effects of nitrogen addition treatments; however, these effects should be considered in future studies (Wang et al., 2019). By considering both biotic and abiotic effects on the microbial community, the influence of climate warming might be better understood and the mechanisms of microbial community in response to warming could be clearly demonstrated in alpine grasslands.

In summary, our work provided evidence that the prokaryotic and fungal communities responded differently to climate warming along the strata profile in alpine grassland and the underlying ecological processes might be helpful to explain such difference. The decreased β-diversity of the microbial community between two soil strata under elevated temperature condition might indicate the vulnerability of ecosystem structure and function to disturbance. Homogeneous selection and dispersal limitation played essential roles in the assembly processes of the prokaryotic and fungal communities, respectively, and both led to a similar community structure under warming conditions. More importantly, the variance of ecological process might be associated with soil heterogeneity, suggesting that the soil heterogeneity may mediate or affect the microbial assembly in response to climate warming as well. By exploring the microbial community structure change along the strata profiles, this study showed the importance of strata in microbial community and deepened the understanding of microbial assembly mechanism in a warmer world.

## Data Availability Statement

The datasets presented in this study can be found in online repositories. The names of the repository/repositories and accession number(s) can be found below: https://nmdc.cn/resource/genomics/project, NMDC10017898&lt.

## Author Contributions

GL and YD developed the original research plan. GL, ND, and YW setup and implemented all site experiments. ZWa implemented sampling, DNA extraction, and 16S rDNA and ITS rDNA sequencing. ZWa and KF did data analysis and wrote the manuscript with help from HY, SW, ZWe, and YD. All authors contributed the intellectual input, assistance to this study, manuscript preparation, and approved the submitted version.

## Conflict of Interest

The authors declare that the research was conducted in the absence of any commercial or financial relationships that could be construed as a potential conflict of interest. The reviewer KL declared a shared affiliation, with no collaboration, with several of the authors ZWa, KF, and ZWe to the handling editor at the time of the review.

## Publisher’s Note

All claims expressed in this article are solely those of the authors and do not necessarily represent those of their affiliated organizations, or those of the publisher, the editors and the reviewers. Any product that may be evaluated in this article, or claim that may be made by its manufacturer, is not guaranteed or endorsed by the publisher.

## Footnotes

1
http://mem.rcees.ac.cn:8080

2
https://nmdc.cn/resource/genomics/project
