# Supplementary material for: Homogeneous Selection and Dispersal Limitation Dominate the Effect of Soil Strata Under Warming Condition
Source: Front Microbiol. 2022 Feb 24;13:801083. doi: 10.3389/fmicb.2022.801083 (PMC8908236; doi:10.3389/fmicb.2022.801083)
Supplement: Supplementary file 1 [file Data_Sheet_1.docx]

**Homogeneous selection and dispersal limitation eliminate the effect of soil strata under warming condition**

**Supporting information**

**
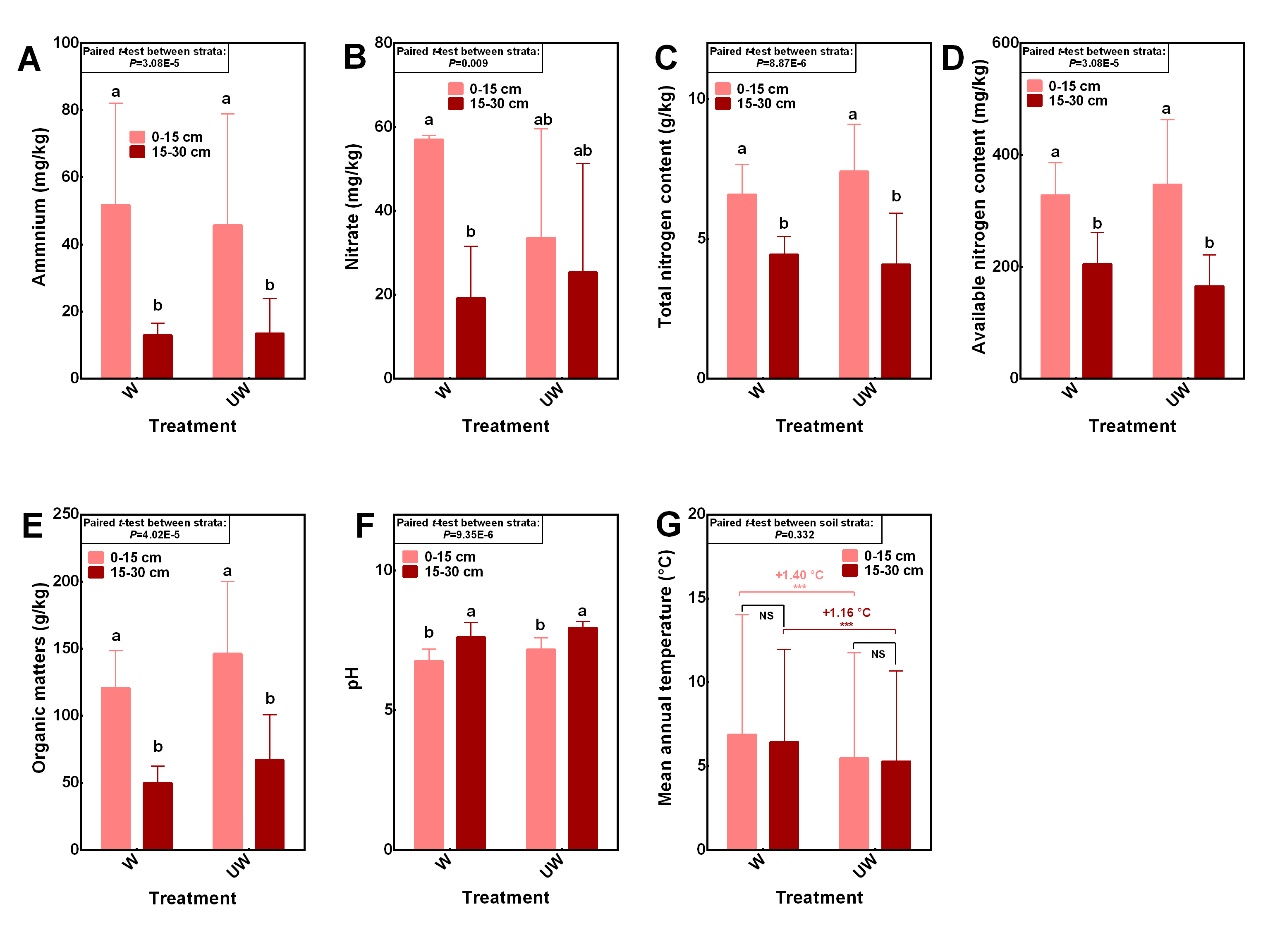
**

Figure S1 Effects of treatments on soil properties. (A) ammonium content (mg/kg); (B) nitrate content (mg/kg); (C) total nitrogen content (g/kg); (D) available nitrogen content (mg/kg); (E) organic matters (g/kg); (F) pH value; (G) mean annual temperature (°C). Different lowercase letters indicate the significance among treatments at *P*<0.05 level by one-way ANOVA. Error bars represent standard deviation. ***: *P*<0.001; NS: not significant, based on paired *t*-test.


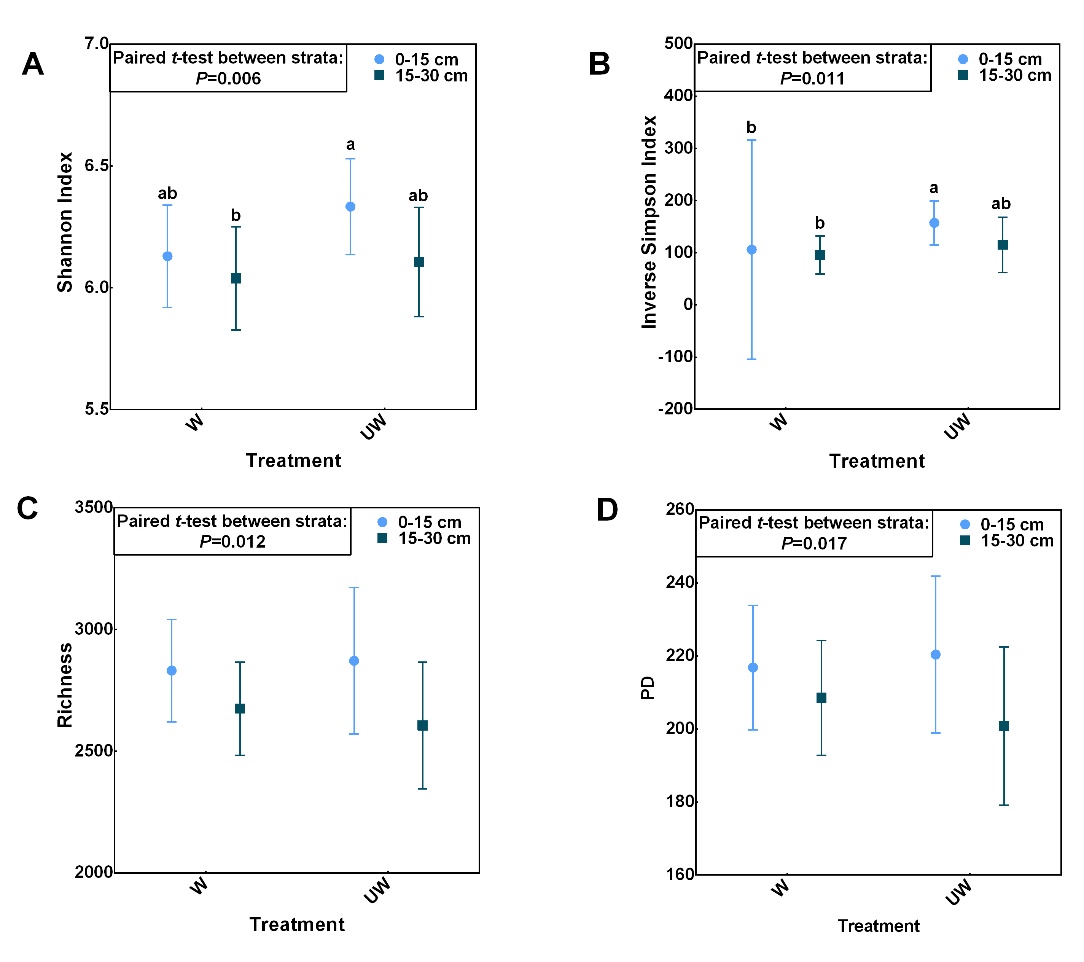


Figure S2 Alpha diversity of the prokaryotic community based on 16S rDNA amplicon sequences. (A) Shannon index; (B) Inverse Simpson index; (C) Richness; (D) PD. Different lowercase letters indicate the significance among treatments at *P*<0.05 level by one-way ANOVA. Error bars represent standard deviation.


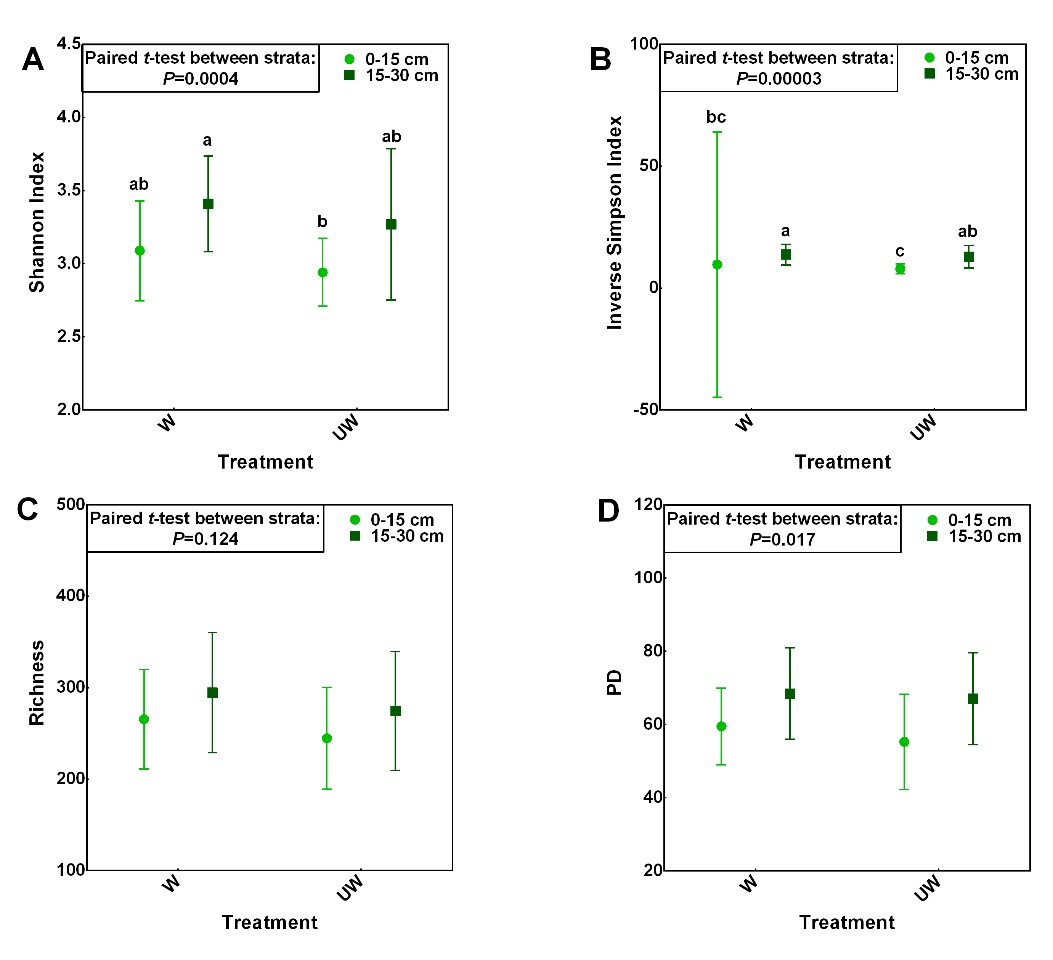


Figure S3 Alpha diversity of the fungal community based on ITS rDNA amplicon sequences. (A) Shannon index; (B) Inverse Simpson index; (C) Richness; (D) PD. Different letters indicate the significance among treatments at *P*<0.05 level by one-way ANOVA. Error bars represent standard deviation.


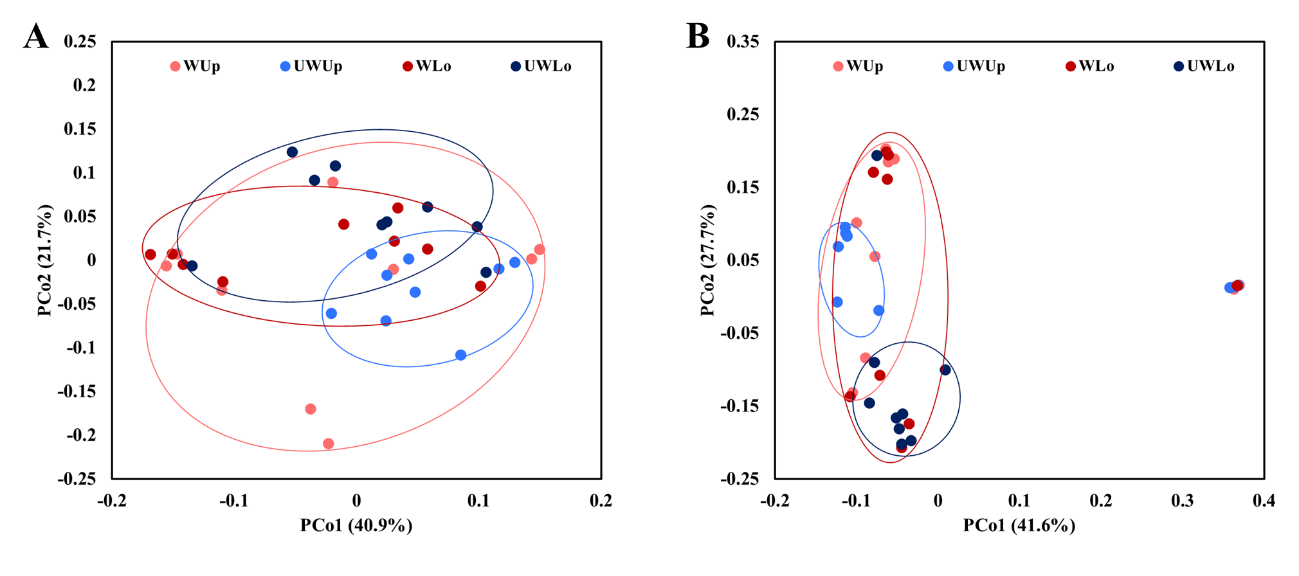


Figure S4 Principal Coordinate Analysis (PCoA) based on 16S rDNA gene amplicon sequences (**A**) and ITS rDNA gene amplicon sequences (**B**). UWUp: unwarming upper; UWLo: unwarming lower; WUp: warming upper; WLo: warming lower.


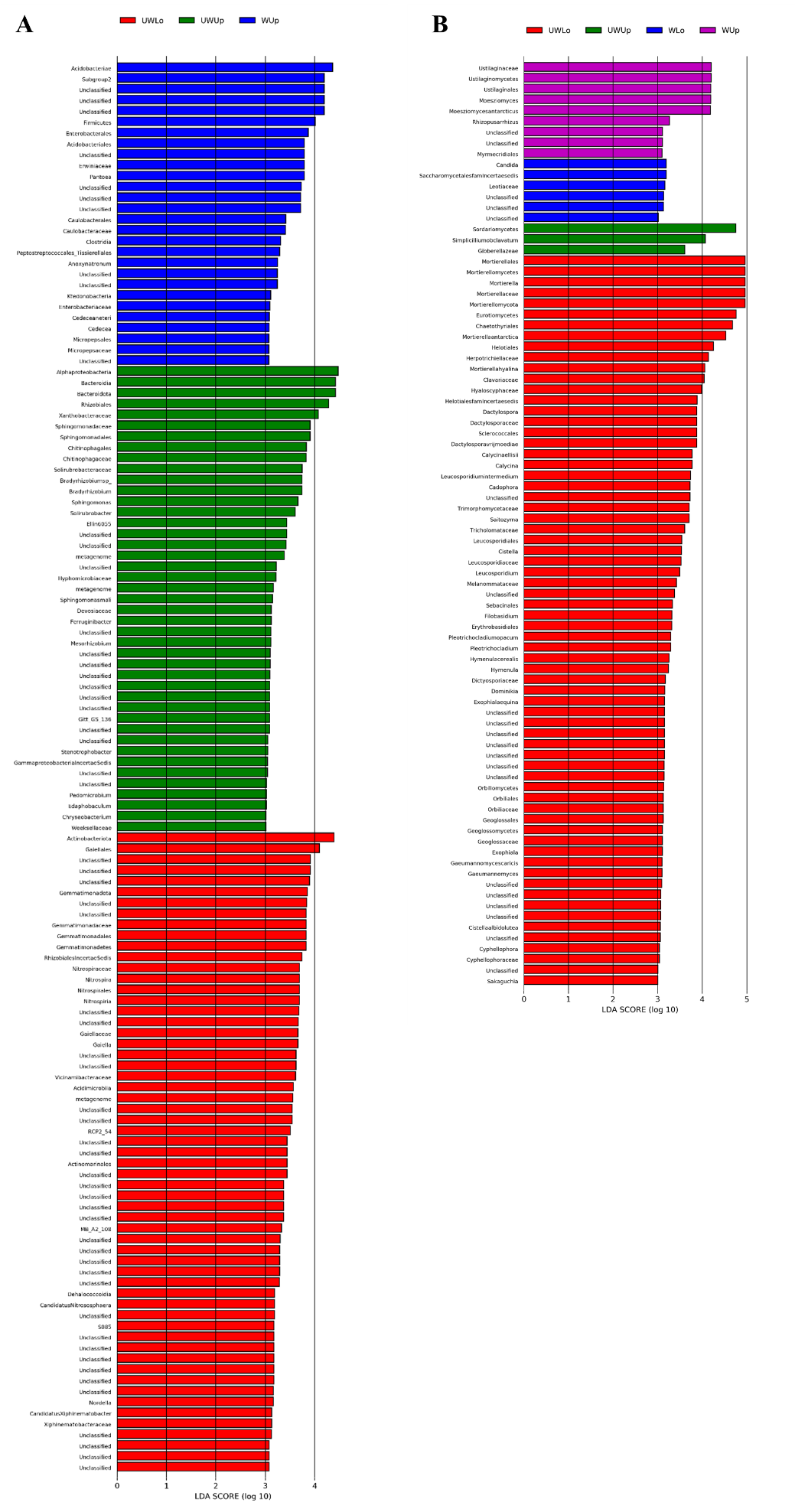


Figure S5 Linear discriminant analysis effect size (LEfSe) identified the most differentially abundant taxa among treatments (*P* < 0.05; LDA score 3.0) based on 16S rDNA gene amplicon sequences (**A**) and ITS rDNA gene amplicon sequences (**B**). LEfSe scores can be interpreted as the degree of consistent difference in relative abundance between features in the different classes of analyzed microbial communities. The histogram thus identifies which clades among all those detected as statistically and biologically differential explain the greatest differences between communities. UWUp: unwarming upper; UWLo: unwarming lower; WUp: warming upper; WLo: warming lower.


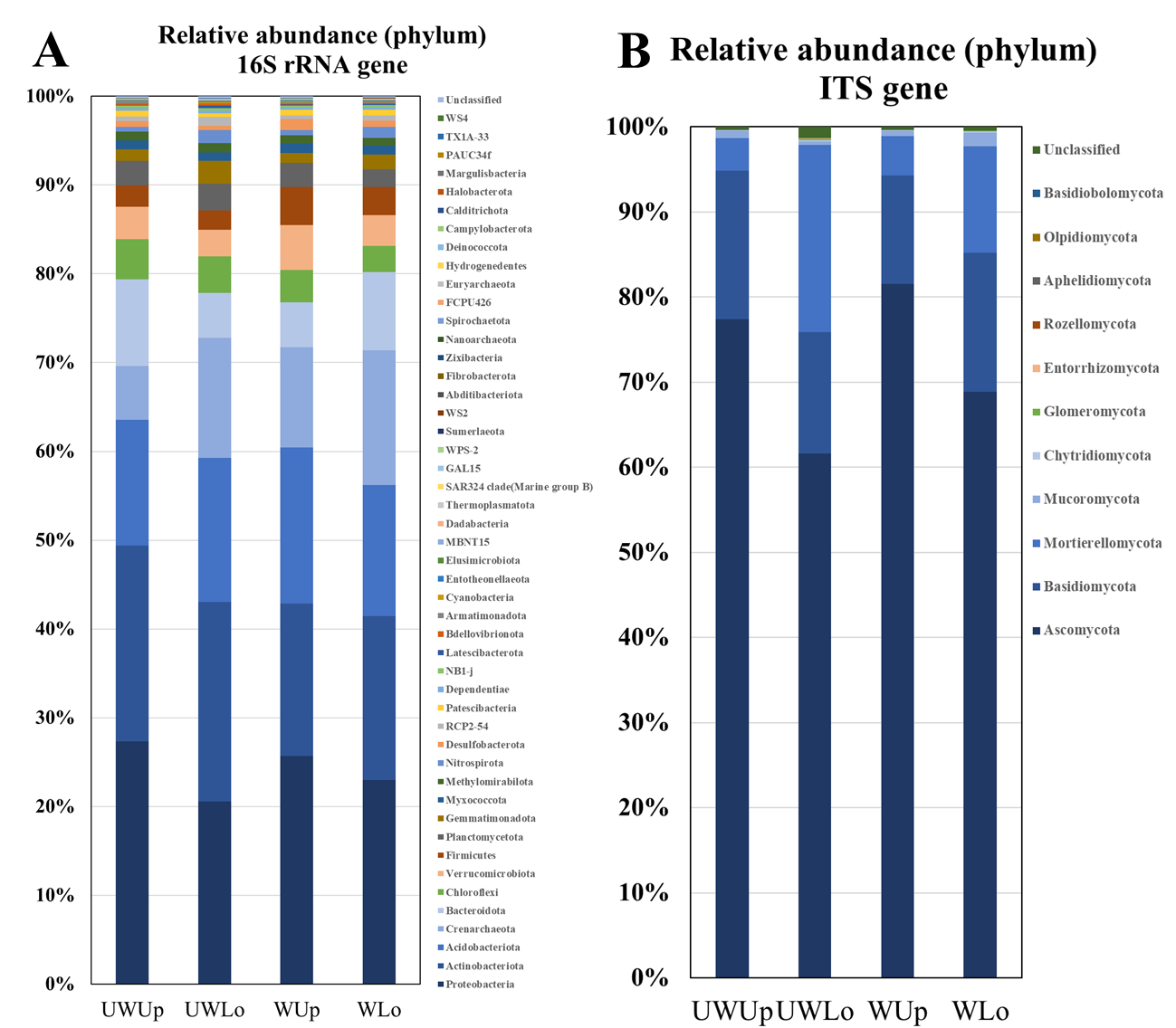


Figure S6 Relative abundance at phylum level based on 16S rDNA gene amplicon sequences (**A**) and ITS rDNA gene amplicon sequences (**B**). UWUp: unwarming upper; UWLo: unwarming lower; WUp: warming upper; WLo: warming lower.


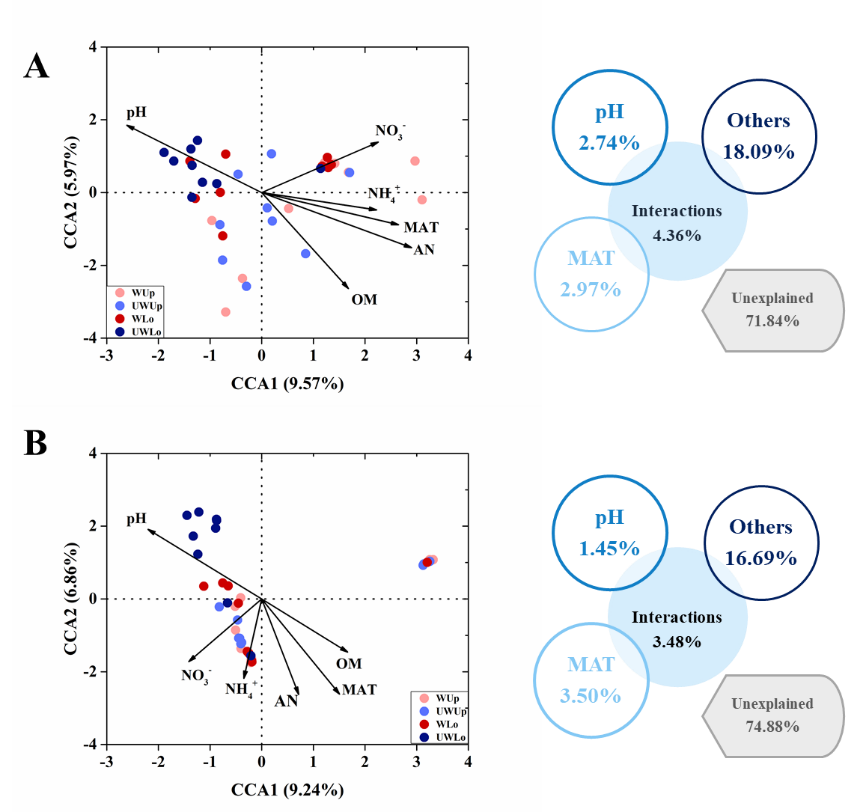

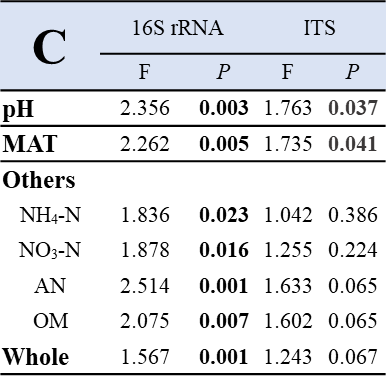


Figure S7 Canonical correspondence analyses (CCA) and variation partitioning analysis (VPA) based on 16S rDNA gene amplicon sequences (**A**) and ITS rDNA gene amplicon sequences (**B**), and model significance of CCA (**C**). UWUp: unwarming upper; UWLo: unwarming lower; WUp: warming upper; WLo: warming lower. NH_4_-N, ammonium content (mg/kg), NO_3_-N, nitrate content (mg/kg). TN, total nitrogen content (g/kg), AN, available nitrogen content (mg/kg), OM, organic matters (g/kg), MAT, soil mean annual temperature.


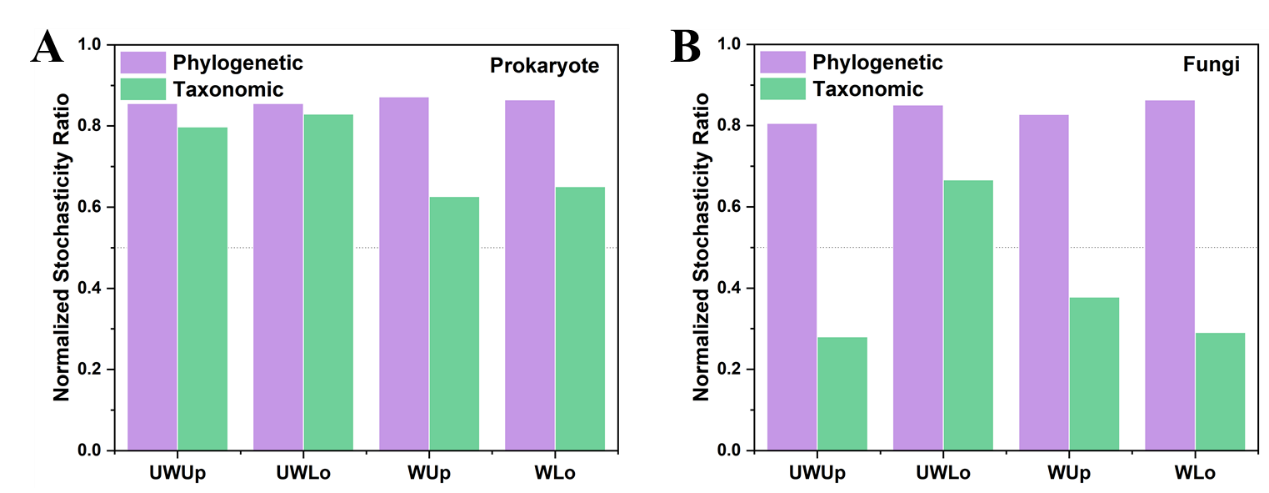


Figure S8 The normalized stochasticity ratio for prokaryote (A) and fungi (B) community based on phylogenetic and taxonomic profiles. UWUp: unwarming upper; UWLo: unwarming lower; WUp: warming upper; WLo: warming lower.

Figure S9 The importance of each phylogenetic bin in the prokaryotic community with associated taxa composition and relative abundance. ***, *P*<0.001, **, *P*<0.01, *, *P*<0.05, showed the significance of the dominating process in each bin based on 1000 bootstrapping times. UWUp: unwarming upper; UWLo: unw
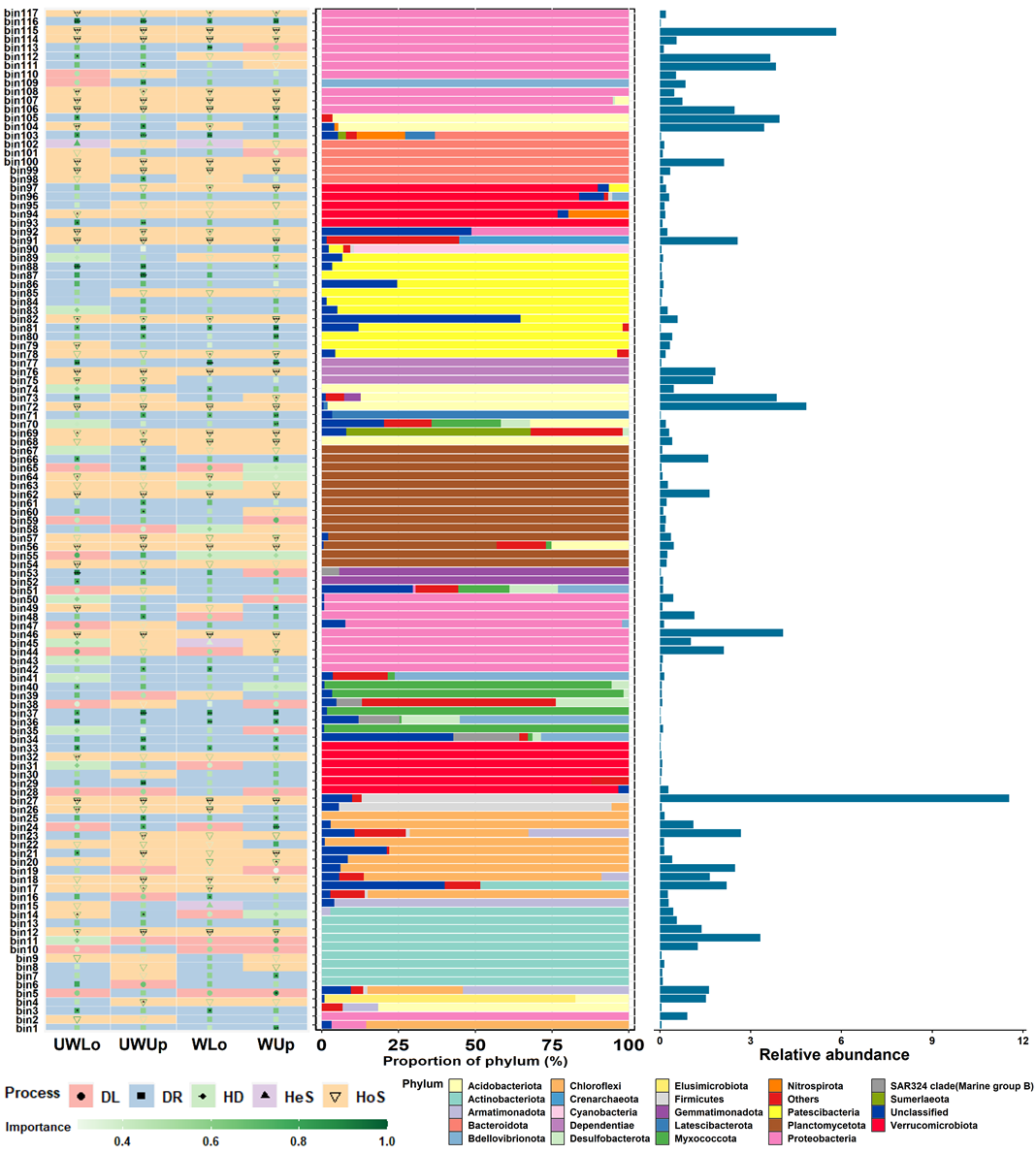
arming lower; WUp: warming upper; WLo: warming lower. DL: dispersal limitation; DR: undominated; HD: homogeneous dispersal; HeS: heterogeneous selection; HoS: homogeneous selection.

Table S1 Effects of warming, strata, and their interaction on microbial communities.

| Dissimilarity test | Warming | | Strata | | Warming & Strata | |
| --- | --- | --- | --- | --- | --- | --- |
|  | F | *P* | F | *P* | F | *P* |
| 16S rRNA gene abundance | 2.6923 | **0.006**** | 3.2128 | **0.005**** | 0.9699 | 0.3870 |
| ITS genes abundance | 1.4914 | 0.187 | 2.7352 | **0.032*** | 0.8187 | 0.512 |

***, *P*<0.001, **, *P*<0.01, *, *P*<0.05, based on permutational multivariate analysis of variance (PERMANOVA).

Table S2 Mantel test for ecological processes with environmental variables for Prokaryote community.

|  |  | HoS | DL |
| --- | --- | --- | --- |
| UWUp | NH_4_-N | -0.255 (0.134) | **0.514 (0.00133)** |
|  | NO_3_-N | 0.251 (0.14) | -0.108 (0.532) |
|  | TN | **-0.459 (0.00481)** | 0.0894 (0.604) |
|  | AN | -0.191 (0.265) | 0.176 (0.305) |
|  | OM | **-0.461 (0.00463)** | 0.0702 (0.684) |
|  | pH | 0.203 (0.235) | 0.123 (0.474) |
| UWLo | NH_4_-N | 0.221 (0.196) | -0.313 (0.0634) |
|  | NO_3_-N | 0.326 (0.0527) | -0.225 (0.186) |
|  | TN | **0.493 (0.00223)** | -0.175 (0.308) |
|  | AN | **0.364 (0.029)** | -0.0749 (0.664) |
|  | OM | **0.489 (0.00249)** | -0.173 (0.312) |
|  | pH | 0.142 (0.409) | 0.121 (0.484) |
| WUp | NH_4_-N | -0.0587 (0.734) | 0.0948 (0.582) |
|  | NO_3_-N | **-0.374 (0.0247)** | **0.334 (0.0462)** |
|  | TN | 0.313 (0.0627) | **-0.349 (0.0371)** |
|  | AN | -0.0736 (0.67) | -0.00448 (0.979) |
|  | OM | 0.241 (0.157) | -0.271 (0.11) |
|  | pH | -0.013 (0.94) | 0.0987 (0.567) |
| WLo | NH_4_-N | 0.0695 (0.687) | -0.0432 (0.802) |
|  | NO_3_-N | -0.0881 (0.61) | -0.0331 (0.848) |
|  | TN | -0.143 (0.406) | 0.318 (0.0585) |
|  | AN | 0.0718 (0.677) | 0.152 (0.376) |
|  | OM | -0.177 (0.302) | **0.409 (0.0132)** |
|  | pH | **0.358 (0.032)** | -0.306 (0.0692) |

The *r* and *P* values shown in bold are at 0.05 significance level. UWUp: unwarming upper; UWLo: unwarming lower; WUp: warming upper; WLo: warming lower.

Table S3 Mantel test for ecological processes with environmental variables for Fungi community.

|  |  | HoS | DL |
| --- | --- | --- | --- |
| UWUp | NH_4_-N | 0.0553 (0.749) | -0.0488 (0.777) |
|  | NO_3_-N | 0.141 (0.414) | -0.0193 (0.911) |
|  | TN | -0.212 (0.215) | 0.141 (0.413) |
|  | AN | 0.0037 (0.983) | -0.0674 (0.696) |
|  | OM | -0.164 (0.339) | 0.117 (0.495) |
|  | pH | 0.105 (0.541) | 0.0777 (0.653) |
| UWLo | NH_4_-N | 0.29 (0.0863) | **-0.341 (0.0419)** |
|  | NO_3_-N | 0.224 (0.189) | -0.219 (0.199) |
|  | TN | 0.168 (0.328) | -0.244 (0.152) |
|  | AN | 0.137 (0.425) | -0.144 (0.401) |
|  | OM | 0.162 (0.345) | -0.234 (0.169) |
|  | pH | -0.0709 (0.681) | 0.0394 (0.819) |
| WUp | NH_4_-N | 0.156 (0.363) | -0.146 (0.395) |
|  | NO_3_-N | -0.103 (0.551) | 0.105 (0.54) |
|  | TN | 0.218 (0.201) | -0.189 (0.27) |
|  | AN | -0.228 (0.181) | 0.0934 (0.588) |
|  | OM | 0.0249 (0.885) | -0.134 (0.435) |
|  | pH | 0.208 (0.224) | -0.137 (0.425) |
| WLo | NH_4_-N | 0.094 (0.585) | -0.0258 (0.881) |
|  | NO_3_-N | 0.129 (0.454) | -0.00565 (0.974) |
|  | TN | -0.0463 (0.789) | 0.318 (0.0584) |
|  | AN | -0.131 (0.445) | 0.244 (0.151) |
|  | OM | -0.117 (0.497) | **0.421 (0.0105)** |
|  | pH | 0.145 (0.4) | -0.123 (0.474) |

The *r* and *P* values shown in bold are at 0.05 significance level. UWUp: unwarming upper; UWLo: unwarming lower; WUp: warming upper; WLo: warming lower.
